# Hemorrhage Control of Liver Injury by Short Electrical Pulses

**DOI:** 10.1371/journal.pone.0049852

**Published:** 2013-01-08

**Authors:** Yossi Mandel, Guy Malki, Eid Adawi, Elon Glassberg, Arnon Afek, Michael Zagetzki, Ofer Barnea

**Affiliations:** 1 IDF Medical Corps, Ramat Gan, Israel; 2 Department of Bio-Medical Engineering, Tel Aviv University, Tel Aviv, Israel; 3 Sheba Medical Center, Ramat Gan, Israel; University of Cambridge, United Kingdom

## Abstract

Trauma is a leading cause of death among young individuals globally and uncontrolled hemorrhage is the leading cause of preventable death. Controlling hemorrhage from a solid organ is often very challenging in military as well as civilian setting. Recent studies demonstrated reversible vasoconstriction and irreversible thrombosis following application of microseconds-long electrical pulses. The current paper describes for the first time reduction in bleeding from the injured liver in rat and rabbit model *in-vivo*. We applied short (25 and 50 µs) electrical pulses of 1250 V/cm to rats and rabbit liver following induction of standardized penetrating injury and measured the amount of bleeding into the abdominal cavity one hour post injury. We found a 60 and 36 percent reduction in blood volume in rats treated by 25 µs and 50 µs, respectively (P<0.001). Similar results were found for the rabbit model. Finite element simulation revealed that the effect was likely non-thermal. Histological evaluation found local cellular injury with intravascular thrombosis. Further research should be done to fully explore the mechanism of action and the potential use of short electric pulses for hemorrhage control.

## Introduction

Trauma is a leading cause of death among young individuals globally. Uncontrolled non-compressible hemorrhage is the leading cause of preventable deaths [1], [2], [3], [4]. Most of the battlefield hemorrhages are compressible, e.g. they can be controlled by tourniquets, haemostatic dressings or other means of direct pressure application. A recent US Army survey demonstrated increased survival rate due to early tourniquet application, while causing minimal collateral damage [5]. In contrary, controlling hemorrhage in the junctional regions (axilla, groin or neck) remains a major challenge in the battlefield and new devices are currently under evaluation [6]. Similarly, truncal hemorrhages, originating from internal cavities (such as chest, peritoneal or retroperitoneal spaces) or solid organs (e.g. liver, spleen, and kidney) are considered as non-compressible and application of effective direct external pressure is not applicable. Controlling hemorrhage from a solid organ may be challenging even in anoperation setting due to the rich vasculature and the lack of supportive connective tissue. Several techniques for controlling hemorrhage from solid organs are being studied to address this major challenge. High Intensity Focused Ultrasound (HIFU) [7], [8] induces a rapid tissue temperature increase and cavitation formation, both leading to thrombosis and platelets activation [9]. This technique causes adverse reactions such as irreversible destruction of the liver and the blood vessels. Another potential treatment for liver injury is the intra-operative application of homeostatic bandages which were demonstrated to be effective in controlling liver hemorrhage in large animal models [10]. Nevertheless, these techniques, as well as others [11], [12], are still under evaluation and have not yet been proven to offer a satisfactory solution to the clinical needs, especially as they all require surgical exposure of the bleeding site.

Long application of direct current has been demonstrated to cause thrombosis of a clamped blood vessel [13] [14] [15]. However, applying this technique for clinical use in hemorrhage control is not practical due to the high risk of the expected injury to the tissue. Short (sub-ms) electrical pulses have been shown to induce constriction of blood vessels and, at higher settings, thrombosis, with minimal thermal effects [16], [17], [18]. The strength-duration dependence of the thresholds of vasoconstriction and thrombosis have been recently measured for the arteries in veins in chicken embryo [19]. That study also determined that the onset time of vasoconstriction was about 10 seconds after the beginning of electrical stimulation, while thrombosis was achieved at approximately 3 minutes.

Analyzing injury data from recent conflicts, a US Army paper [20] estimated that “Effective methods for the treatment of non-compressible penetrating truncal injury will have the greatest potential to impact DOW (Died Of Wound) and KIA (Killed In Action) rates in current overseas contingency operations. The pre-hospital arena offers the best opportunity for impact.” We believe that the technique describe in this paper has the potential to be applied in pre-hospital and hospital care for management of non-compressible hemorrhage. Our long term goal is to develop a portable device to control internal non-compressible hemorrhage from solid organs. The current paper describes for the first time that sub-millisecond electric pulses reduce the amount of bleeding in a liver injury. We also demonstrate that the temperature rise during the treatment is minimal, indicating that treatment mechanism is likely non-thermal. Histological analysis of treated liver further shows the thrombotic effect of electrical pulses

## Results

### Blood Loss

**Figure 1:**
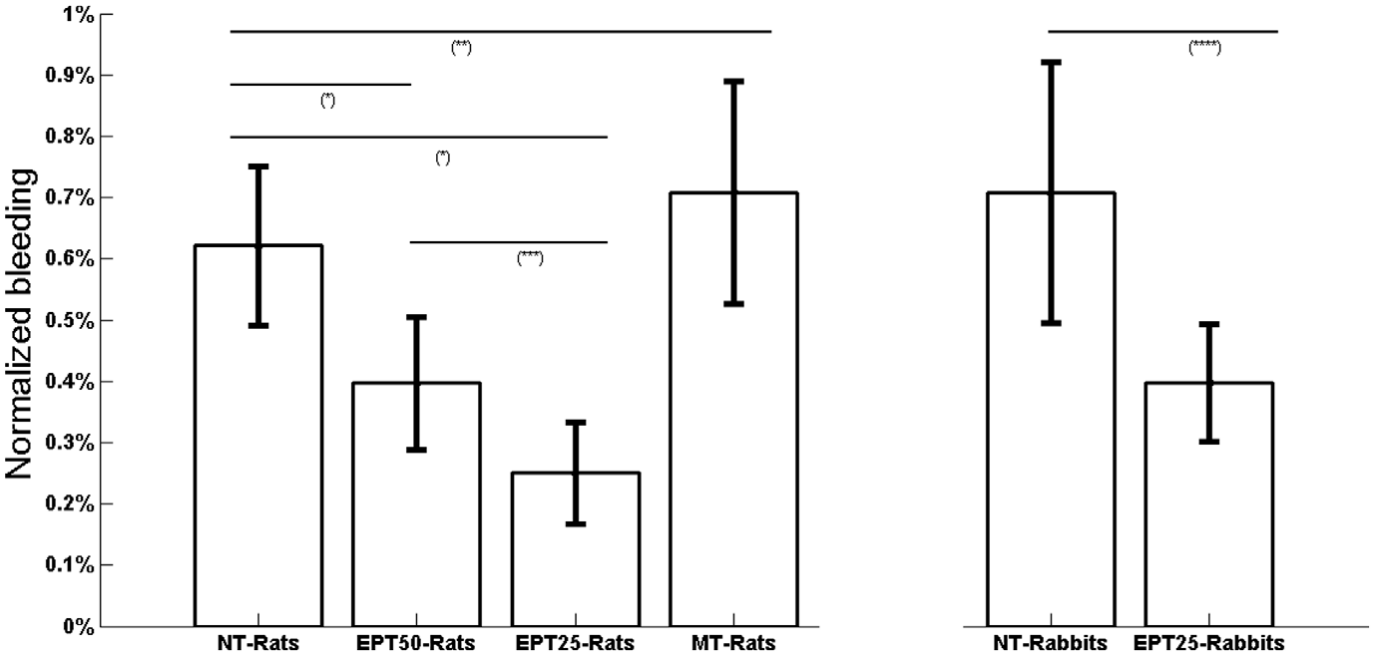
Experimental setup of rat (a) and rabbit (b) liver. (a) Injured rat liver (long arrows) was treated with two parallel plate electrodes (short arrows) mounted on a hand caliper adjusting for liver thickness. (b) Rabbit liver injury was treated by fix parallel plates electrodes (7.2 mm apart) (short arrows) positioned at two sides of the wound (dotted line).

Body weight, excised liver weight and normalized excised liver weight were not significantly different between four animal groups (p>0.1) (**Table 1**). In contrast, blood loss in rats treated with 50 µs (EPT-50) and 25 µs (EPT-25) was significantly reduced by 36% and 60%, respectively, as compared to the non-treated (NT) control group (p<0.001 for both groups, **Table 1** **,** **Fig. 1**). Blood loss in the EPT-25 group was significantly lower than in the EPT-50 group (p = 0.025). Blood loss in the mechanical pressure (MP) group did not differ significantly from the NT group (p = 0.43). Similar results were found for rabbits (**Table 1** **,** **Fig. 1**), where blood loss in the electric pulse treatment group was reduced by more than 40 percent compared to the non-treatment group (p = 0.004).

**Table 1:** Animal weight, excised liver weight, absolute and normalized blood loss in all animal treatment groups.

	Rat			Rabbit		
	No treatment (NT)	Mechanical Pressure (MP)	50 µs pulses (EPT-50)	25 µs pulses (EPT-25)	25 µs pulses (EPT-25)	No Treatment (NT)
N	11	4	8	5	7	7
**Animal weight G**	398.66±20.14	408.3±49.5	398±32.9	392.3±33.6	3196±448	3106±293
**Normalized Excised Liver Weight ±SD %**	0.26±0.07	0.25±0.06	0.22±0.05	0.21±0.05	NA	NA
**Absolute blood loss ±SD G**	2.48±0.57±	2.83±0.65	1.58±0.41	0.97±0.3	21.66±5.54	12.63±3.31
**Normalized blood loss ±SD G**	0.62±0.13	0.71±0.18	0.4±0.11	0.25±0.08	0.40±0.1	0.71±0.21

### Bio Heat Model

**Figure 2:**
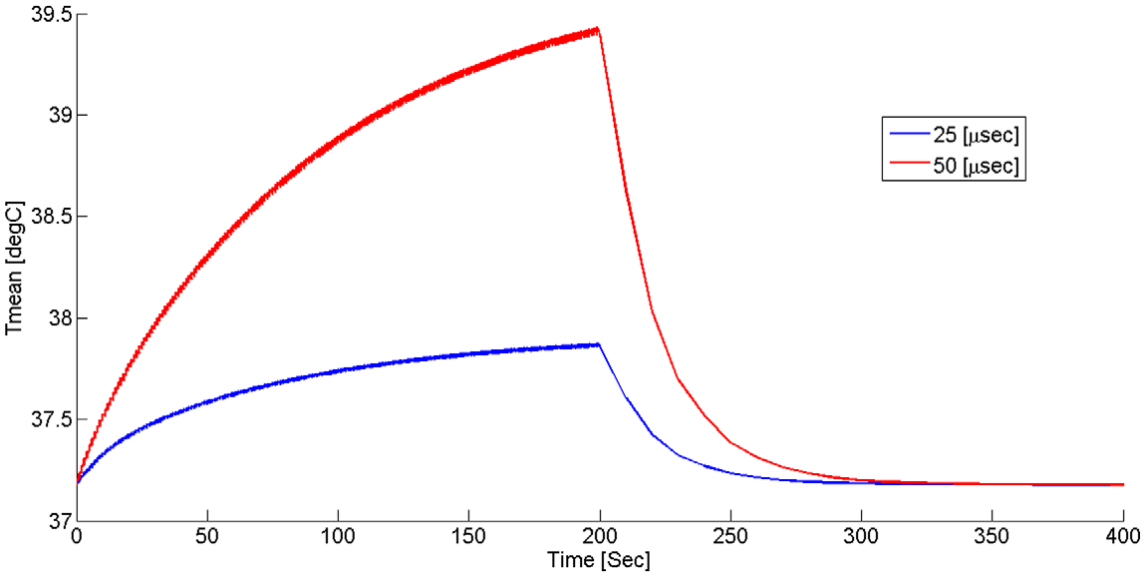
Normalized bleeding weight in all animal groups (rats and rabbits). Control groups were not treated. EPT50 and EPT 25 were treated by 200 pulses of 50 and 25 µs, respectively, in a repetition rate of 1 Hz. Bars represents standard error. Unpaired t-test results for various comparisons are as follows: (*) p<0.001, (**) p = 0.43, (***) p = 0.025, (****) p = 0.004.

**Figure 3:**
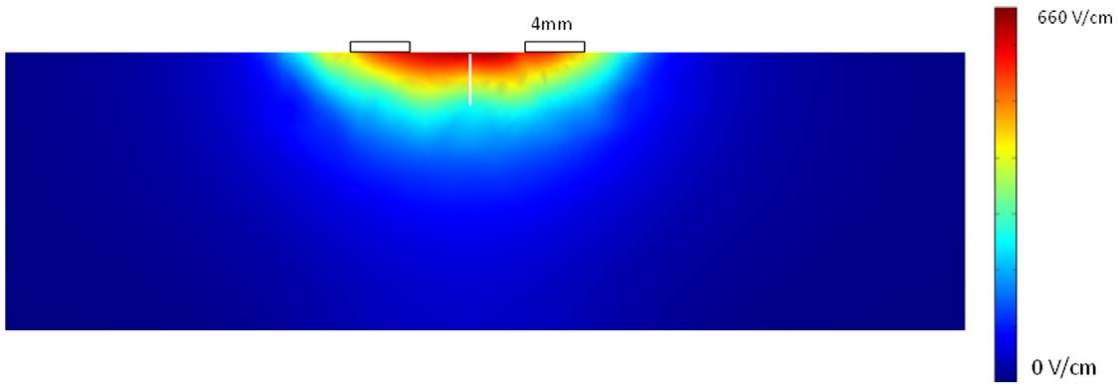
Calculated mean temperature versus time of rat liver treated by 200 pulses of 25 and 50 µs at pulse potential of 500 V with repetition rate of 1 Hz. Following a mild increase in temperature, there is a rapid relaxation of temperature to baseline.

Solution to the heat generation and conduction in tissue (**Fig. 2**) showed a mild temporal increase in liver temperature in response to 200 electric pulses. Maximal liver temperature following treatment was 39.4°C with 50 µs pulses and 37.9°C with 25 µs pulses. The temperature increase was transient, and returned to normal after about 100 second following treatment. Solution to the electric problem in the rabbit electrode configuration revealed that the electrical field below the electrode was relatively uniform with maximal value of 660 V/cm (**Fig. 3**). The electric field decreased along the depth of the tissue to 213 V/cm at the lower point of the liver cut. The heat solution for the rabbit treatment showed a minimal temperature elevation of less than 0.15°C at the described treatment protocol.

Measurements of temperature with a thermal camera in two rabbits treated with 200 pulses of 500 Volts showed that immediately after treatment the average surface temperature below the electrodes was only mildly increased (by 0.2 and 1.04°C ; initial temperature were 34.3 and 35.04°C, respectively). The mild increase in temperature is in good agreement with the bio heat solution for the rabbit case. Importantly, our bio-heat model, as well as similar published experiments (e.g. [21]) predict that maximal electrical field and temperature increase are expected to occur at the tissue surface close to the treatment electrode. The thermal camera also measures surface temperature, and therefore the small temperature increase found in liver surface temperature assess the maximum temperature rise in tissue.

### Histology

**Figure 4:**
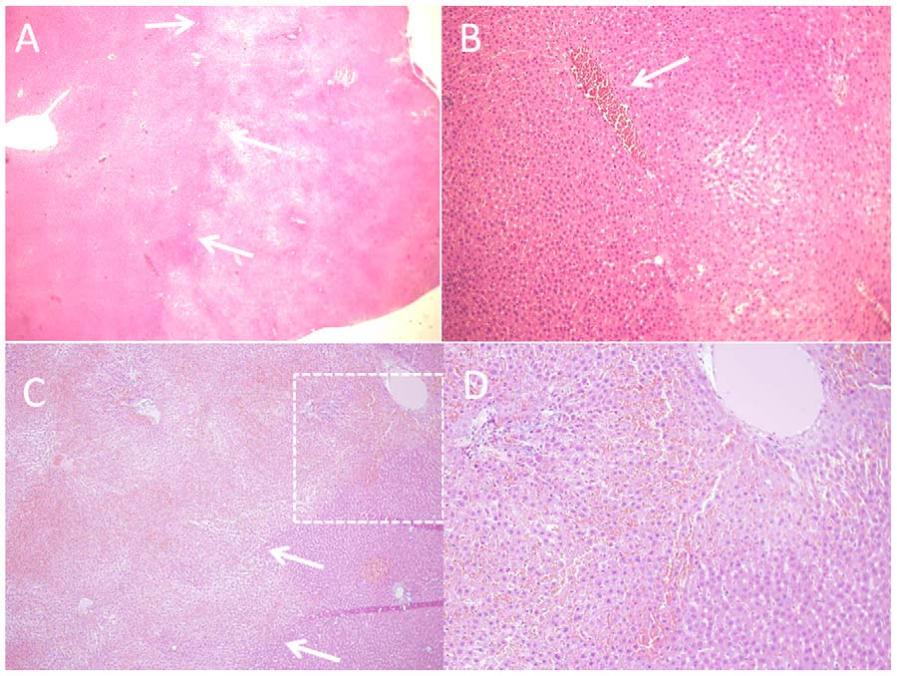
Simulation results of electric field around the liver cut (white line) following pulsing with 4 mm electrodes spaced apart by 7.2 mm.

We analyzed six histological specimens from 4 rats and 2 rabbits. **Fig. 4** shows characteristic histological results of rat (A,B) and rabbit (C,D) livers treated with short electrical pulses. **Fig. 4** **A** shows a sharp demarcation line between normal and affected areas (arrows) in rat liver treated with 200 pulses of 50 microseconds with electrical field of 1250 V/cm. Higher magnification of the same preparation shows hepatocytes with eosinophilic discoloration, unclear cells borders and red blood cells clogging in large blood vessels, which might be the mechanism of hemorrhage control by electric pulses (**Fig. 4** **B**). Rabbit liver treated with 100 pulses of 500 volts at pulse duration of 25 µs shows similar changes as in the rat liver with distinct border between treated area (left to arrows) to untreated area (**Fig. 4** **C**). Higher magnification of the same area depicts eosinophilic cellular discoloration, picnotic nuclei in many of hepatocytes and extensive extravasation of RBC's due to clogging of blood vessels. Unaffected area looks normal at the right lower corner of the figure looks normal (**Fig. 4** **D**).

## Discussion

Our results demonstrate that short electrical pulses decreased the bleeding volume from liver injury by 60 and 44 percent for rats and rabbits, respectively (**Fig. 1** **,** **Table 1**). The bleeding volume was not different between the NT group and the MP group in rats, suggesting that the treatment effect was not caused by the mechanical pressure applied by the electrodes per-se, but rather by the electric field applied on the tissue. Another potential explanation for the reduction of the hemorrhage could be thermal coagulation induced by increased temperature caused by Joule heating. However, thermo coagulation is usually expected at temperature of above 60–70°C [22], [23], which according to the mathematical simulation (**Fig. 2**) and the experimental measurements, were not achieved even for the longer pulse duration (50 µs). Further, there was no evidence for thermo coagulation in the histological sections (**Fig. 4**).

Another potential mechanism for hemorrhage reduction could be associated with blood vessels contraction, caused by smooth muscle activation. The liver blood flow is controlled by a pressure-flow auto regulation mechanism, which is mediated by changes in the blood vessel's muscle tonus as well as other mechanisms such as the contraction of stellate cells in response to various physiological stimuli [24], [25], [26]. Although the main branches of the hepatic and portal arteries do contain a muscular layer, it is sparely found in the more distal branches [26]. Taken together, it seems that the role of vascular contraction in the electrical induced hemorrhage control is still unclear.

We hypothesize that the hemorrhage control observed in this study is associated to endothelial layer damage, leading to thrombosis and irreversible vessel constriction. Similar effect were reported in other studies of electrochemical therapy [27]. Ramirez *et al.* [28] reported that electrical pulses of 850 V/cm, 100 microseconds long, caused a decrease in blood perfusion to the spleen and mesenteric arteries. The authors reported that electrical pulsing of the liver caused a decrease in perfusion, as was demonstrated by a color test. About 20 minutes after pulsing, the perfusion gradually returned to normal. Sersa *et al.* [29] found that 3 minutes following electric pulsing of tumors, blood flow decreased by about 80 percent and histological evaluation of the endothelial cells showed that they were rounded up and swollen causing narrowing of blood vessels lumen. Eight hours after pulsing apoptosis was found in some vessels as well as extravasations and stacking of erythrocytes. The effect on blood vessels is believed to be related to immediate disruption of microfilament and microtubule cytoskeleton, liquid extravasations, increased interstitial fluid pressure and blood vessel collapse [30]. The author suggested that the effect of smooth-muscle-induced-vasoconstriction was probably less important in the case of tumor blood vessels, which have small amount of smooth muscles [27]. Palanker *et al.* [19] described both reversible and irreversible effects of electric pulsed on blood vessels. Histological examination revealed damage to blood vessels endothelium, which might be the reason for both reversible vasoconstriction and irreversible thrombosis. Possible cause for this endothelial damage is irreversible electroporation (IRE), which is caused by irreversible nano-size pore formation in the cellular membrane [27], [31], [32]. The effect was probably non-thermal as the temperature rise during the treatment did not exceed 0.01°C. The authors reported that the threshold voltage for reversible vasoconstriction was higher for arteries (80 V) than for veins (60 V), and did not depend on the size of the vessels. However, the threshold of thrombosis increased with the vessel diameter.

Interestingly, we found that 25 microsecond pulses were significantly more effective in reducing hemorrhage volume in rats as compared to 50 microsecond pulses (**Fig. 1**). Possible explanation could be associated with a local increase in liver perfusion in response to mild temperature rise in the case of 50 microsecond pulses. (**Fig. 2**). Previous studies showed that a local increase in liver temperature to 43°C caused a local increase in blood perfusion [33]. We hypothesize that such a local increase in perfusion could cause a relative increase in blood loss, partially reducing the effect of treatment. For this reasons we used 25 µs pulses in the rabbit model.

In parallel to the haemostatic effect of the high electric field pulse treatment, it can cause unwanted effect on normal liver cells, as was found in histological preparation (**Fig. 4**). The results are similar to previously described pathological changes following irreversible electroporation in normal liver tissue [21], [34], and sarcoma tumors [35]. These changes are probably caused by direct effect of electric field on cells and are not a thermal effect. The later could be caused only in instances where temperature was above 50°C for at least 3 minutes, which is not the case, according to our simulation. The FE simulation estimated the electric field in the area of interest in the rabbit case to be between 660 to 213 V/cm which partially overlap with irreversible electroporation thresholds. Thus, there is a potential damage effect of the short electrical pulses on the liver. However, as opposed to thermal based ablation (e.g. RF or cryo-ablation), irreversible electroporation does not induce changes in connective tissue [36], [37], [38], [39], [40] enabling enhanced regenerative process of the liver [41]. Nevertheless, this potential adverse effect calls for future studies aiming an reducing the damage by changing electrode configuration or treatment protocol and parameters.

Our study has several limitations to be considered. First, blood pressure and pulse were not measured or controlled during the experiments. This could theoretically increase variance in amount of bleeding. Nevertheless, the standard variations in bleeding within the various treatment groups were low and, even without adjustment to blood pressure, were significantly different. Second, in this preliminary study we did not addressed the effect of treatment in the case of traumatic coagulopathy, which is expected in cases of severe liver trauma. Other issues to be studied in future research in larger animals are the design of electrodes in order to optimize electric field geometry, pulse parameters, better control of tissue temperature and the possible use of changes in tissue electrical properties for measuring treatment effect. Of great importance and an engineering challenge is to explore the possibility to apply the electrical field by non-invasive methods. This approach is used for few applications such as transcranial magnetic stimulation [42] or cardiac stimulation [43]. However, the electrical field induced for this application is about 2–3 orders smaller than required for the current application.

In conclusion, in this preliminary research we demonstrate that short electric pulses can significantly reduce the amount of bleeding from injured liver in a rat and rabbit model. The effect is non-thermal and possibly related to direct effect on blood vessels endothelial layer. Further research is needed in order to optimized treatment protocol and to fully expose possible potential uses for hemorrhage control in both civilian and military settings.

## Materials and Methods

### Rats Liver Injury Protocol

Twenty eight adult male Sprague-Dawley rats were used during the experiments. The experiment protocol was approved by the Animal Rights Council of the Israel Ministry of Health and conformed to guidelines for the humane care of animals. Rats and rabbits were supplied by Harlan Laboratories Ltd., Jerusalem, at the age of 3 months. Average animal weight is depicted in Table 1. Animals were anesthetized with an intra muscular injection of Ketamine HCI (0.19 ml/100 gram) and Xylazine (0.03 ml/100 gram) solution. Following injections, the rats were placed on heating blankets and observed for full anesthesia. Additional anesthetics were given approximately every 20 minutes by titration. Twenty minutes following the administration of anesthetic drugs, a midline abdominal incision was performed and the liver was gently exposed. The median lobe of the liver was resected 13 mm from lobe edge and the removed part was weighed by portable scales (Ohaus Company, model N2B110) and is referred in text as “excised liver weight”. This weight was further normalized to animal total weight.

**Figure 5:**
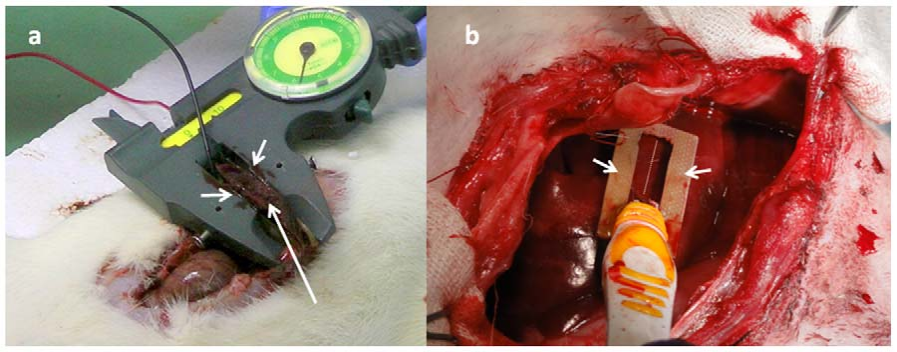
H&E staining of rat (A,B) and rabbit (C,D) livers treated with short electrical pulses following injury. Figure A shows a sharp demarcation line between normal and treated areas (arrows) in rat liver treated with 200 pulses of 50 microseconds with electrical field of 1250 V/cm. B Higher magnification of the same. C. Rabbit liver treated with 100 pulses of 500 volts at pulse duration of 25 µs shows similar changes as in A,B with distinct border between treated area (left to arrows) to untreated area. D. Higher magnification of the same area depicts eosinophilic cellular discoloration, picnotic nuclei in many of hepatocytes and extensive extravasation of RBC's due to clogging of blood vessels.

Following liver injury, rats were divided into four groups. Electrical pulse treatment group (EPT50), received 200 electrical pulses of 500 volts with pulse duration of 50 µs at pulse repetition rate of 1 Hz. EPT25 group received 200 similar pulses given at pulse duration of 25 µs. The medial liver lobe of rat was placed between two customized copper electrodes attached to a commercial caliper (**Fig. 5a**). The distance between the 2 parallel slabs was adjustable, and determined for each animal by the liver thickness (Mean+SD electrode distance 3.98±0.56 mm). A series of electrical pulses generated by Square Wave Electroporation System (ECM 830, Harvard Apparatus), operating in the mono-phasic mode was applied to the EPT-50 and EPT-25 groups. Thus, the mean pulsed electric field (E = V/d) was about 1250 V/cm.

Treatment regimen was evaluated based on preliminary experiments and literature on liver [21] and blood vessel [31] electroporation. For control we used two groups: no treatment (NT) group and mechanical pressure (MP) group, in which the electrodes were placed on the median lobe, similar to the EPT groups for 200 seconds, however, no pulses were delivered.

Following these interventions, the abdomen was closed using continuous sutures and the rats were maintained on a heated blanket for 1 hour without any further treatment. Total blood loss was measured 60 minutes after liver injury by soaking a cotton wool in the peritoneal cavity, as reported by previous authors [44], [45], [46], [47]. Blood loss for each animal was normalized by its body weight. All surgical intervention and measurements were performed by the same investigator (YM).

### Rabbit Liver Injury Protocol

Fourteen adult male New Zealand rabbits were used during the experiments. Rabbits were supplied by Harlan Laboratories Ltd., Jerusalem. Average animal weight is depicted in Table 1. Animals were anesthetized with an intra venous injection of Ketamine HCI (50 mg/kg) and Xylazine (3.5 mg/kg) solution followed by maintenance dosage. Twenty minutes following the administration of anesthetic drugs, a midline abdominal incision was performed, the liver was gently exposed and 3 cuts 5 mm deep and 3 cm long were performed in the three liver lobes (total 9 cuts).

Following liver injury, rabbits were divided into two groups. EPT-25 group received 200 pulses that were given with pulse duration of 25 and NT group received no treatment. In EPT-25 group rabbit liver was treated with customized electrodes comprised of two copper plates 4 mm wide, 29 mm long and spaced apart by 7.2 mm (**Fig. 5b**). Following each liver cut, the electrodes were positioned on the liver surface while the cut is in equal distances between the electrodes and a series of electrical pulses were given, similarly to the treatment described for rat liver injury.

Following these interventions, rabbit's abdomen was closed and the animals were maintained on a heated blanket for 1 hour without any further treatment. Total blood loss was measured 60 minutes after liver injury in the same method as described for rats. All surgical intervention and measurements for all experiments were performed by the same investigators (YM, GM). The pulse wave form was recorded with an oscilloscope (Tektronix TDS 220) during treatment in three rabbits. The measured waveform followed a square voltage pulse (with rise time of less than 1 µs and fall time of about 1 µs) and reached the target voltage at a good accuracy (of 95–99 percent of the desired pulse potential).

### Finite Element Analysis and Temperature Measurement

The thermal effects of the electrical pulses in the liver were estimated by finite element analysis of the electrical field and the resulting heat field using Comsol Multiphysics 3.5 software (COMSOL inc., Sweden). The method was previously reported by our group [48]. Rat liver was modeled as a box with dimensions of 3.5×6×0.5 cm, and with 3.5×0.5 cm rectangular electrode, simulating the real dimension of the electrodes. Rabbit liver lobes were modeled as a 2×8×8 cm box where electrodes were modeled with the real dimensions of the treatment electrodes (see Material and Methods and **Fig. 4**). The electric potential and field for each spatial point in the liver were calculated by the solution of Poisson's equation. Electrodes were represented as surfaces of a fixed-voltage boundary condition in which one electrode had a positive potential V_p_ referred to as the *pulse potential* and the other was zero (the ground). The outer boundaries of the box were set to an insulating condition and a continuity condition was applied to all other boundaries. After solving the field (Poisson) equation, the Joule heating (*p*) rate per unit volume (W/m^3^) caused by the electric field can be calculated by:
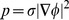
(1)were σ is tissue condictivity and φ is the electric potential. The Joule heat generated during the electroporation treatment was then added to the Pennes bio-heat equation [49] (Eq. 2) when pulse is on and set to zero when pulse is off. The following equation was solved for different scenarios:
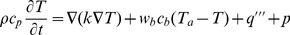
(2)where *k* is the thermal conductivity of the tissue, *T* is the temperature, *w_b_* is the blood perfusion, c_b_ is the heat capacity of the blood, *T_a_* is the arterial temperature, *q‴* is the metabolic heat generation, *p* is the electric heat generation, *ρ* is the tissue density, and *c_p_* is the heat capacity of the tissue. The boundary conditions for the bio-heat model were divided into two subdomains, one within the body while the other was protruding out of the body cavity during treatment, exposed to the surrounding environment at 25°C with heat flux. The simulation calculate separately the resistive heating and temperature rise during pulse interval and the temperature relaxation during time interval between pulses. The problem was solved separately for each time interval while using the solution at the end of one time interval as the initial condition for the next one. These steps were repeated according to the number of pulses. Values used for the simulation analysis are summarized in Table 2. Following an optimization process of runtime and accuracy, body meshed was done with 2304 tetrahedral elements.

**Table 2:** Parameters used for numerical study.

Parameter	Symbol [units]	Value	Reference
Tissue Electrical Conductivity	σ [S/m]	0.286	[50]
Thermal Conductivity	k [W/(m·k)]	0.512	[50]
Tissue Density	ρ [Kg/m^3^]	1050	[50]
Tissue Heat Capacity	C_p_ [J/(kg·k)]	3600	[50]
Blood Density	ρ_b_ [Kg/m^3^]	1000	[51]
Blood Heat Capacity	C_b_ [J/(kg·k)]	3640	[50]
Blood Perfusion Rate	ω_b_ [1/s]	1e-3 or 6.4e-3	[52]
Blood temperature	T_b_ [k]	310.15	-
Heat Transfer Coefficient	h [W/(m2·k)]	10	[53]
Surrounding Temperature	T_s_ [k]	298.15	-

Surface temperature of two rabbit livers was measured in vivo by ThermoVisionÂ A40 infrared camera (FLIR systems, USA) immediately following pulse treatment. The camera was positioned 50 cm from the liver and acquired thermal pictures at autofocus mode. Thermal pictures before and after measurements were exported to Matlab 2011b and the average temperature of liver surface below the electrode was calculated before and after treatment.

### Histology preparation

The liver was removed immediately after euthanasia (on average 87 minutes following treatment) and fixed into formaldehyde 10%. Histological slices were processed for H&E staining in paraffin sections and then cut perpendicular to liver edge in order to demonstrate the transition between treated and untreated zones.
