# Correction: Hemorrhage Control of Liver Injury by Short Electrical Pulses

**DOI:** 10.1371/annotation/2eb01d65-7384-4397-b1f0-40ce5e515953

**Published:** 2013-08-08

**Authors:** Yossi Mandel, Guy Malki, Eid Adawi, Elon Glassberg, Arnon Afek, Michael Zagetzki, Ofer Barnea

The titles and legends of multiple figures were accidentally switched. The title and legend currently appearing with Figure 1 belong with Figure 5, the title and legend currently appearing with Figure 2 belong with Figure 1, the title and legend currently appearing with Figure 3 belong with Figure 2, the title and legend currently appearing with Figure 4 belong with Figure 3, and the title and legend currently appearing with Figure 5 belong with Figure 4. 

